# Long Term Positive Effect of Grassland Restoration on Plant Diversity - Success or Not?

**DOI:** 10.1371/journal.pone.0155836

**Published:** 2016-05-19

**Authors:** Emelie Waldén, Regina Lindborg

**Affiliations:** Department of Physical Geography, Stockholm University, Stockholm, Sweden; Institute of Tibetan Plateau Research, CHINA

## Abstract

Restoration is important for biodiversity conservation worldwide, but surprisingly little is known about its efficiency in a long-term perspective. In this study, we re-examined Swedish semi-natural grasslands 12–20 years after the restoration, by comparing field inventories of vascular plant species diversity made in 2001 with follow-up inventories in 2012. We also analysed restoration effect in relation to six environmental factors and used continuously managed semi-natural grasslands as references of desired state after restoration. We found that total species richness increased over time but not to reference levels, while there were no significant changes in species density or number of grassland specialists. However, the overall species composition in the restored sites, as well as grassland specialist composition, now largely resembled reference conditions. Fertilisation and time between abandonment and restoration were the only environmental variables that affected total species composition change, while site area affected change in grassland specialist composition. Our results show that restoration of semi-natural grasslands can contribute to conservation of semi-natural habitats and their associated biodiversity. Yet, due to the vague restoration goals for these sites, it is difficult to evaluate the restoration success, which emphasise the general need for clear and measurable goals.

## Introduction

Humans have altered practically every natural ecosystem on the Earth [1,2], some to the point of collapse. To mitigate this process, large resources are invested in conserving natural habitats and restoring degraded and damaged ecosystems [3–5]. There are many different kinds of ecological restoration, ranging from rehabilitation of industrial fields and mines, to improving biological values in production landscapes [6]. Another aspect of restoration is the creation of new natural areas on former intensively used areas, for example habitat creation on post-mining or landfill sites (e.g. [7,8]), or creation of pastures on former agricultural fields (e.g. [9–12]). The latter is a common restoration method in European rural landscapes. However, this should not be confused with restoration of degraded but not fundamentally altered habitats, such as restoration of former semi-natural grasslands (cf.[13]) by tree clearing and re-introducing grazing, which is the focus of this study.

The societal benefits of ecological restoration in farmlands have mostly been discussed in terms of enhancement of biodiversity and ecosystem services [3,14,15]. Since semi-natural grasslands are important habitats within farmlands for both biological and cultural reasons, economic compensation to conserve and restore them is a central part of agri-environmental schemes (AES) in many European countries [16]. A major drawback acknowledged in many restoration studies is the lack of measurable goals, which causes difficulties in defining restoration ‘success’ [17–19] and evaluating the outcome of a particular restoration scheme [20]. For semi-natural grassland restoration, aims can vary from pure biodiversity preservation [21–23], to the preservation of aesthetic values [21] or the promotion of traditional values or historical conditions [22,24,25]. The restoration outcome is often measured by increased overall species richness and occurrence of grassland specialists or rare species, but both general guidelines (e.g. [26]) and research studies (e.g. [27,28]), suggest that a species composition similar to reference grasslands is a better measurement of success. This is a fairly common measure to evaluate success when creating new grasslands on former arable fields (e.g. [10]), but only a few multi-site studies have evaluated long-term effects of restoration of degraded semi-natural grasslands [27,29,30].

Plant species richness in semi-natural grasslands has been shown to increase with elapsed time since restoration (e.g. [27,30,31]). In some studies, the grassland plant community has recovered 3–12 years after restoration ([28,32], but see e.g. [22,33]). Richness of grassland specialists rarely recovers fully following restoration (but see [34]), although increasing trends after 3–15 years have been found [30,35,36]. Similarily, increasing trends have been shown regarding species density on a m^2^-scale [28,37]. Although species composition may partly recover after restoration [38], it is often significantly different from reference grasslands [22,30,39,40], even after 12–15 years ([27,34], but see [29]).

Several factors are important for the successful outcome of semi-natural grassland restoration [22,41,42]. Site area, as well as partial shrub and tree cover have been found to positively affect species diversity [43–45], while vegetation height has a negative effect [46]. During semi-natural grassland abandonment, a gradual succession towards broad-leaved forests begin, leading to a decline in grassland specialist species [47–49]. Long-overgrown grasslands have been found to have lower semi-natural grassland potential than set-aside agricultural fields [50,51], indicating the negative effect of the time between abandonment and restoration. Another factor negatively affecting species richness is fertilisation, since high nutrient concentration is a constraining factor for plant diversity [37,52,53].

One of the priorities in the EU Rural Development Programme is the so-called *High Nature Value* farming, where conservation of biodiversity is maintained by low intensity farming, with semi-natural grasslands as a key feature [54,55]. Restoration of these has occurred for several decades, but relatively few studies has evaluated the outcome [4,23], especially over long time periods [28]. In this study, we analyse 16 Swedish semi-natural grasslands 12–20 years after the restoration, by analysing changes in vascular plant species richness and composition. We examined 1) how richness, density (number of species/m^2^) and composition for both all plants and grassland specialist species changed since restoration, 2) the similarities between restored and reference sites, and 3) how the environmental factors; restored site area, time between abandonment of grazing and restoration, time since restoration, abundance of trees and shrubs at the restored site, vegetation height and degree of fertilisation affected potential change in diversity and species composition.

## Methods

### Study areas

We compared data from a plant survey in 2001 of 16 abandoned and restored semi-natural grasslands [22] with a re-inventory eleven years later (2012). All sites are situated in the three counties of Södermanland, Uppland and Östergötland in south-eastern Sweden (57°50′ N to 60°28′ N; 15°10′ E to 18°25′ E). The mean annual precipitation is 550 mm and the mean temperature in summer is 16°C and -3°C in the winter. The dominating bedrock is Precambrian gneiss, but the soils and nutrient levels might differ among the sites. The counties have similar remnants of traditional small-scale agriculture and the abiotic conditions are relatively equal (see [22]), leading to a similar species pool [30]. The sites were chosen in 2001 with acquired information from the County Administrative Boards of Södermanland and Östergötland and by Upplandsstiftelsen. All sites have dry to dry-mesic abiotic conditions and were grazed before abandonment. The restoration method was clearing of trees and shrubs and reintroducing livestock grazing. In Sweden, common restoration practices do not involve any kind of seed sowing or seedling planting, which requires that target species have to be present in the vegetation, in the soil seed bank, or disperse via seed rain from nearby populations [56]. The restoration procedures were usually carefully planned, while the restoration aims were vague or non-existent. Existing goals alluded to increased biodiversity and cultural values, but some also mentioned preserving grassland species, ancient monuments and small-scale agricultural landscapes.

In addition to the 16 restored grasslands, we also surveyed five continuously-managed semi-natural grasslands, which were used as references of the desired state after restoration (hereafter called ‘references’), with respect to plant richness and composition. They were of similar size (average = 6.8 ha, restored sites: average = 7.7 ha) and located in the vicinity to the restored sites. All sites were inventoried with permission from the land owner.

### Data collection

Data collection of plant species richness and abundance followed the same protocol in the restored sites and the reference sites, using ten 1m^2^ plots equally distributed in two 40m transects per site both in 2001 (time 1, T1) and 2012 (time 2, T2). The inventories took place during the summer months July and August both years. It is known that year-to-year weather fluctuations could largely affect the vegetation dynamics (e.g. [57,58]), however none of the inventoried years were particularly extreme in that aspect. In total, six different biodiversity measures were analysed for each site at two different scales; site scale and plot scale. On site scale (1) total number of species recorded in ten 1m^2^ plots (hereafter called ‘total number of species’) and (2) total number of semi-natural grassland specialist species recorded in ten 1m^2^ plots (hereafter called ‘total number of grassland specialists’) were analysed. On plot scale, (3) average number of species/m^2^ (hereafter called ‘species density’), (4) average number of grassland specialists/m^2^ (hereafter called ‘specialist species density’), (5) frequency of species/m^2^, and (6) frequency of grassland specialists/m^2^ were analysed. Grassland specialist species were defined as having their optimum occurrence in traditionally managed meadows and pastures and decrease in frequency in early to intermediate successional phase [59,60].

Six explanatory factors were included in the analysis; (1) restored site area, (2) the time elapsed since restoration started, (3) time between abandonment of grazing and restoration, (4) abundance of trees and shrubs at the restored site, (5) vegetation height and (6) degree of fertilisation. The site areas varied between 3 and 25 ha and restoration started between 12 and 20 years ago (average = 14.8 years). The abandonment time was divided into three groups; (1) sites that had low intensity grazing, insufficient to fully prevent succession, during the last 50 years, (2) sites abandoned (i.e. not grazed) for 10–15 years, and (3) sites abandoned for >15 years. Information about site area, time since restoration and abandonment time were acquired from the farmers and county boards. The number of trees and shrubs were counted within a circle with a radius of 20 m both in 2001 & 2012, but since it did not significantly differ between years (Wilcoxon rank sum-test, p = 0.38), an average (ranging from 1 to 60 trees) for each site was used in the analyses. As a measure of grazing intensity [61], vegetation height was measured in each square-meter plot and an average for each site was calculated prior to analysis. The average vegetation height varied between 2.8–14.7 cm. Information regarding degree of fertilisation was acquired from the farmers of each site (divided into three levels; (0) no added fertilisation, (1) fertilised once but not heavily and (2) fertilised more than once, but not heavily or regularly). More exact data of abandonment time, grazing intensity and degree of fertilisation would have been preferable, but was not accessible from the farmers. The sites are described individually in Table 1.

**Table 1:** Site descriptions of 16 restored permanent semi-natural grasslands (A-P) in Sweden.

Site	X Coordinate	Y Coordinate	Grazers	Area	Time since restoration	Abandonment time	Tree abundance	Vegetation height	Nutrient level
A	585727	6536285	Cattle	5	16	1	18	6.2	1
B	613811	6555006	Sheep	7	NA	NA	6.5	6.2	1
C	631997	6630008	Cattle	20	15	2	21.5	4.8	2
D	634292	6630245	Cattle	8	12	1	17	4.6	1
E	587319	6466949	Sheep	10	14	2	60	8.0	2
F	526517	6420525	Cattle	10	15	1	16	3.5	0
G	632491	6624495	Cattle	1.5	12	3	21.5	5.6	0
H	556161	6428582	Cattle	3	13	1	15.5	14.7	0
I	555872	6428719	Cattle	1.8	13	1	2	7.3	2
J	596519	6550566	Cattle	10	20	1	17	7.3	1
K	524082	6410703	Cattle	25	14	1	0.5	11.2	2
L	586051	6468524	Cattle	4.5	14	1	15	7.2	2
M	625966	6548889	Sheep, Horses	5	18	3	18	8.2	1
N	517103	6420607	Cattle	2	13	3	19	7.1	2
O	611311	6502862	Cattle	7	16	3	11	6.2	1
P	621498	6582263	Cattle	3	17	1	14	2.8	0

Site location (X and Y coordinates, SWEREF99), grazing species, area (hectare), time since the site were restored, time between abandonment and restoration (divided in three groups: (1) sites that had low intensity grazing, insufficient to fully prevent succession, during the last 50 years, (2) sites abandoned (i.e. not grazed) for 10–15 years, and (3) sites abandoned for >15 years), average number of trees and bushes, average vegetation height (in cm) and level of nutrients (divided in three groups: no added fertilisation (0), fertilised once but not heavily (1) and fertilised more than once but not heavily or regularly (2)). Information about site area, time since restoration, abandonment time and nutrient level were acquired from the farmers and county boards.

### Statistical analyses

The statistical analyses were performed using R 2.15.0 [62], CANOCO 5 [63] and PAST 2.17b [64]. All species data were Poisson-distributed and square-root transformed prior analyses. Three of the environmental factors (restored site area, time since restoration and average abundance of trees and shrubs) were log-transformed before further analysis. Changes in biodiversity measures between 2001 and 2012, were analysed in paired t-tests in R (after assuming normality and homogenous variances by F-tests). Differences in biodiversity measures between restored and reference sites were analysed in Welch t-tests in R. To examine whether change in biodiversity measures depended on the environmental factors, a ratio between T1 and T2 (i.e. value year 2012/value year 2001) was calculated for each site and each biodiversity measure, before performing regression analyses in R (linear regression and multiple regression).

The composition of all species and grassland specialists were analysed in CANOCO 5 and PAST, using detrended correspondence analysis (DCA) and analysis of similarity (based on Bray-Curtis distance measure, number of permutations = 9999) to examine if the species composition had changed in the restored sites between T1 and T2, as well as to compare with species composition in the reference sites. Further, constrained partial canonical correspondence analysis (CCA) with ‘Time’ set as a co-variable was used to analyse which environmental factors contributed to the species composition (down-weighted rare species) and grassland specialist composition. One site was not included in these analyses due to missing environmental data. SIMPER-analyses with Bray-Curtis distance measure [65] were used to examine which species were primarily responsible for the observed differences in the species composition in the restored sites between T1 and T2.

## Results

### Total and specialist plant species richness and density

The total number of species increased significantly in the restored semi-natural grasslands between 2001 and 2012, from 51.8 to 62.9 species per site (Table 2). The total number of grassland specialists also increased from 19.3 to 21.3 species/site, but the increase was not significant (p-value = 0.087, Table 2). Hence, the increase in total number of species related more to an increase of generalist species than of specialist species. The species density (average number of species/m^2^) did not differ significantly from T1 to T2, neither did the grassland specialist density (Table 2). None of the explanatory variables; restored site area (log), abandonment time, time since restoration (log), abundance of trees and shrubs (log), vegetation height or fertilisation, could explain the significant change in total number of species, either alone (linear regressions, p>0.05), or all combined (multiple regression, p>0.05).

**Table 2:** Diversity in restored semi-natural grasslands measured at two different time steps.

Biodiversity measure	Average ± 95%CI	Difference in mean T1-T2	Paired t-test (difference between years)	Significance level
Total no. species *-Restored*	T1: 51.81 ± 5.40, T2: 62.94 ± 5.41	+11.13	t = -4.6365, df = 15, p-value = 0.0003	***
No. species/m^2^ *-Restored*	T1: 18.61 ± 2.18, T2: 19.29 ± 1.98	+0.68	t = -0.756, df = 15, p-value = 0.4614	NS
Total no. grassland specialist species *-Restored*	T1: 19.25 ± 3.37, T2: 21.38 ± 3.08	+2.13	t = -1.8296, df = 15,p-value = 0.0873	NS
No. grassland specialist species/m^2^ *-Restored*	T1: 6.71 ± 1.64, T2: 6.37 ± 1.45	-0.34	t = 0.649, df = 15, p-value = 0.5261	NS
Total no. species *-Reference*	T2: 78.00 ± 4.06			
No. species/m^2^ *-Reference*	T2: 27.30 ± 3.01			
Total no. grassland specialist species *-Reference*	T2: 33.80 ± 1.14			
No. grassland specialist species/m2 *-Reference*	T2: 14.02 ± 0.99			

Difference between the years (T1 = 2001, T2 = 2012) tested using paired t-test. Significance level indicated by asterisks (*** p < 0.001, NS = non-significant). Reference semi-natural grassland diversity measured in 2012.

**Fig 1:**
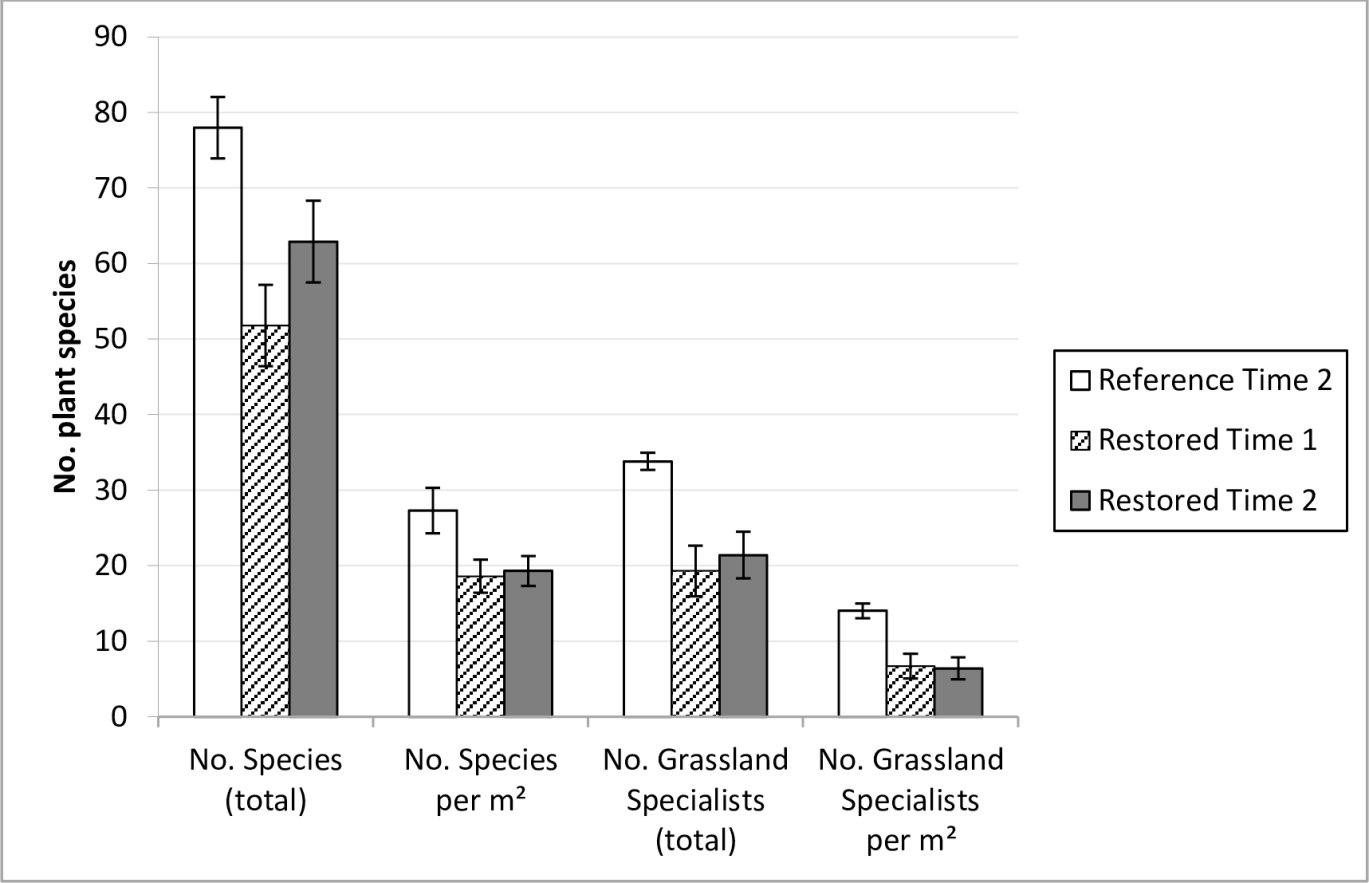
Plant species richness found in restored and reference semi-natural grasslands. Total number of plant species and grassland specialists and per m^2^(95% CI) found in reference semi-natural grasslands at time 2 (i.e. 2012) and restored semi-natural grasslands at time 1 and 2 (i.e. 2001 and 2012).

Even though the total number of species increased in the restored sites, there were still significantly more species in the reference sites, even after the inventory in 2012 (mean difference: 15.06, t = 4.363, df = 16.729, p<0.001). There were also significantly more grassland specialists (mean difference = 12.42, t = 7.418, df = 18.129, p<0.001), a higher number of species per m^2^ (mean difference = 8.01, t = 4.361, df = 7.815, p = 0.003), as well as a higher number of grassland specialists per m^2^ (mean difference = 7.65, t = 8.543, df = 17.783, p< 0.001) in the reference sites than the restored sites (Fig 1). Out of the 239 species recorded in the restored and reference sites in 2012, 36 (including 18 grassland specialists) were unique to the reference sites and 84 (including 18 grassland specialists) were unique to the restored sites (see S1 Appendix).

### Species composition

**Fig 2:**
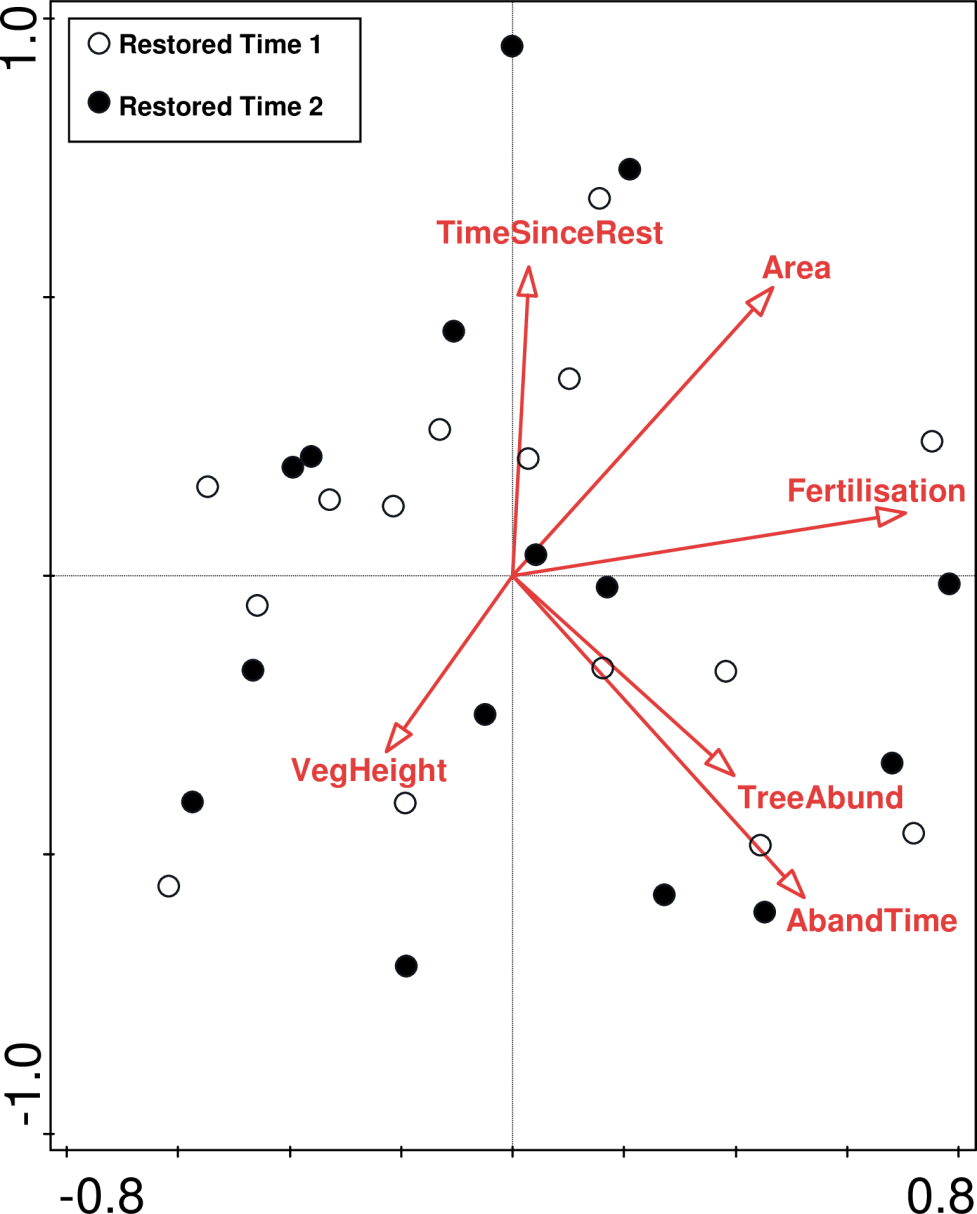
Local variables responsible for the species composition in the restored semi-natural grasslands. Constrained partial canonical correspondence analysis (down-weighting rare species, inventory time set as a co-variable (Time 1 = year 2001, Time 2 = year 2012)). The variables ‘Fertilisation’ (explained 5.2%) and ‘Abandonment time’ (explained 4.9%) were significant.

**Fig 3:**
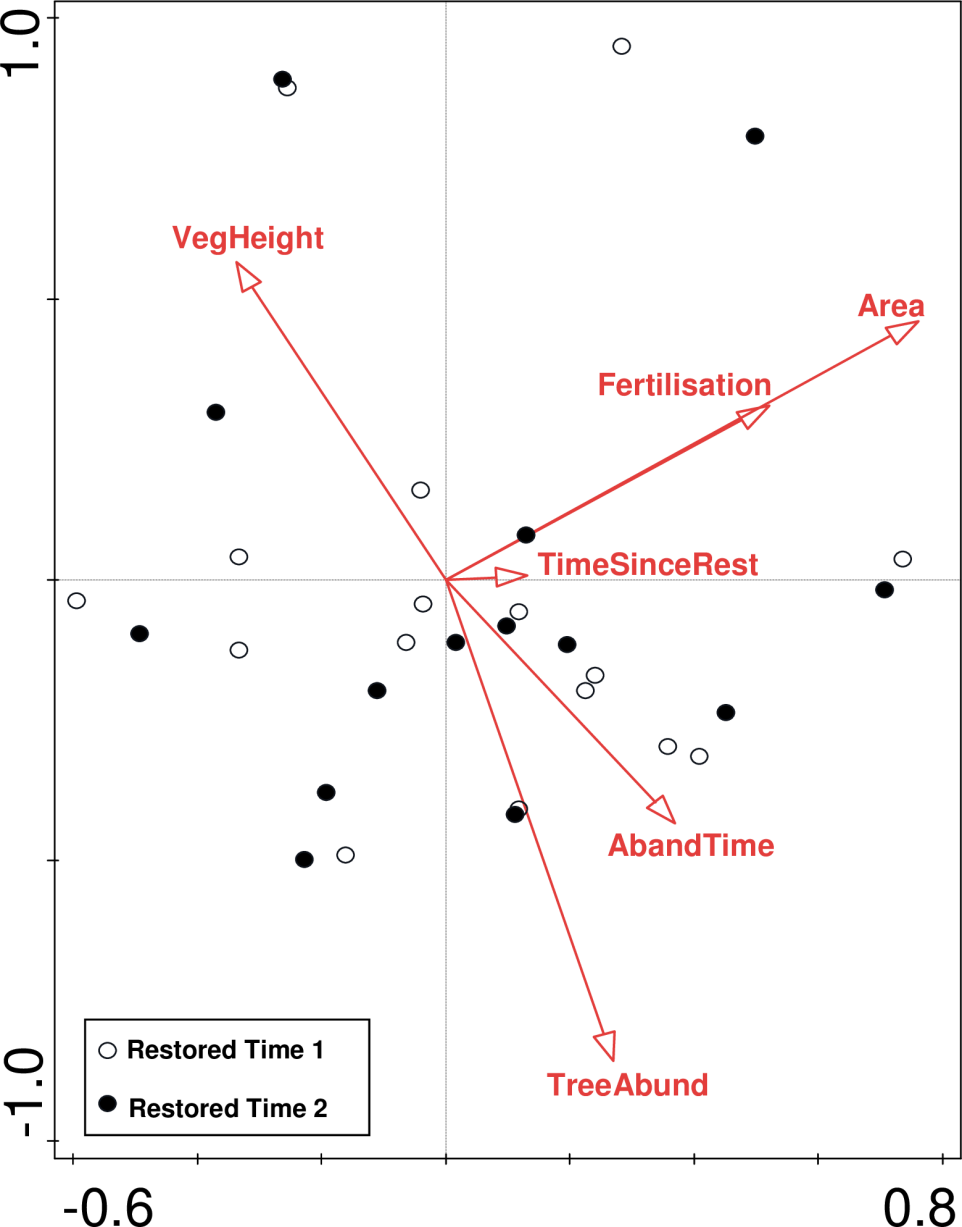
Local variables responsible for the specialist species composition in the restored semi-natural grasslands. Constrained partial canonical correspondence analysis (inventory time set as a co-variable (Time 1 = year 2001, Time 2 = year 2012)). The variable ‘Area (log)’ (explained 5.1%) was significant.

Composition of both the entire community and grasslands specialists only, changed significantly (R = 0.23, p<0.001, and R = 0.1849, p<0.001 respectively) in the restored sites between 2001 and 2012. Out of the 246 species recorded in the restored sites, 146 (including 34 specialists) increased in frequency, while 91 (including 36 specialists) decreased. The species’ contributions to the dissimilarity in species composition were evenly distributed (ranging from 1.57% to 0.05% for total species composition, and from 3.99% to 0.13% for specialist species composition, see S1 Appendix for details). The environmental factors that significantly affected changes in total species composition were ‘Fertilisation’ (explained 5.2%, p = 0.019 and pseudo-F = 1.5) and ‘Abandonment time’ (explained 4.9%, p = 0.037 and pseudo-F = 1.4) (Fig 2), while ‘Area (log)’only contributed significantly to the grassland specialist composition change (explained 5.1%, p = 0.028, pseudo-F = 1.4) (Fig 3). The factors ‘Tree abundance (log)’, ‘Vegetation height’ and ‘Time since restoration (log)’ did not significantly affect overall species composition or grassland specialist composition.

**Fig 4:**
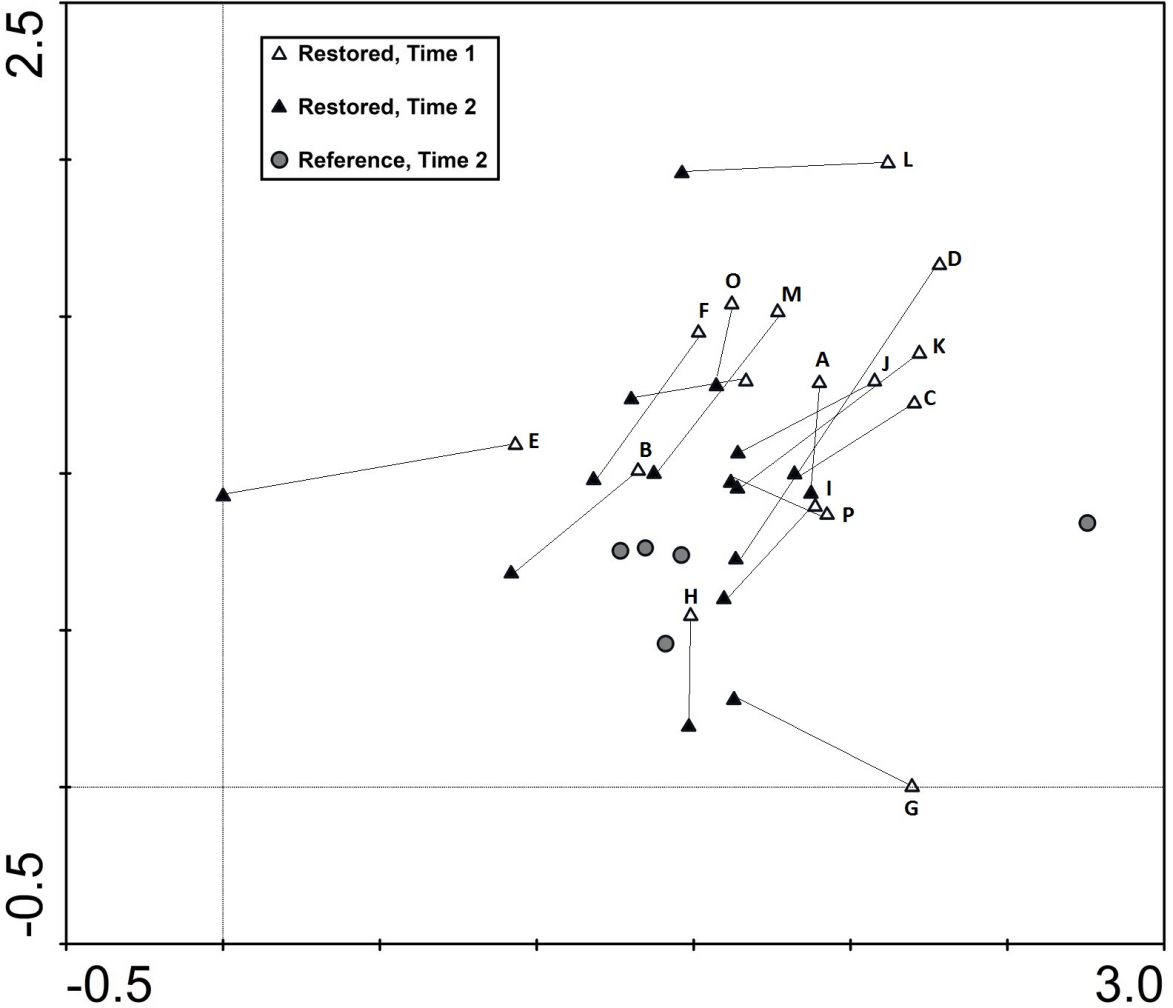
Species composition shift in restored semi-natural grasslands. Detrended correspondence analysis (DCA) of the total species composition in the restored sites at Time 1 (T1, i.e. 2001) and Time 2 (T2, i.e. 2012), and at the continuously managed reference sites at Time 2.

Interestingly, there was no significant difference in either total species composition or grassland specialist composition between the restored sites (at T2) and the continuously managed reference sites (p = 0.375 and p = 0.090, respectively) (Fig 4).

## Discussion

### Species richness and density

Overall, our results showed long-term positive effects of semi-natural grassland restoration on plant diversity, although different diversity measures gave different results. The total number of plant species increased in the restored sites between Time 1 and 2 (i.e. year 2001 and 2012). A similar trend was found regarding the number of grassland specialists. Since we focused on restoration effects both on plant species diversity and composition, the restoration progress in our study sites could partly be described as successful. However, the increased species richness was more related to an increase of generalist species, than of grassland specialists. Furthermore, even though species richness has increased over time, this did not result in an increased species density. Moreover, there were still significant differences between the restored sites and reference sites regarding all the tested species richness and density measures. For instance, there were on average 37% more grassland specialists and more than twice as many specialists per m^2^ in the reference sites, compared to the restored grasslands. This is consistent with the other studies that have compared restored sites with reference sites in a shorter time perspective (i.e. 1–15 years, e.g. [27,30,39]).

Species richness in restored semi-natural grasslands has been found to recover more quickly than species density [66], perhaps because species richness is more sensitive to environmental heterogeneity [28]. A large variety of microhabitats within a grassland could be beneficial for a variety of species [43,44,67], including non-specialists, while species density sometimes is more related to a long continuity of grazing [68]. Congruent with this study, species richness has also been shown to increase with time since restoration [30,37,69], whereas recovery of specialists and rare species has been less successful [28,35,70]. One explanation may be due to the relatively short lifespan of grassland specialists in the seed bank ([71–73], but see [74]). Seed bank persistence is crucial for species re-establishment in restored semi-natural grasslands as seeds are not manually introduced. Another limiting factor could be difficulties for seed dispersal among sites in the fragmented landscape [36,75,76], where grasslands are smaller and more isolated. Further, the establishment capacity of specialized grassland species could also be reduced by unsuitable biotic and/or abiotic factors in the restored sites [27].

### Species composition

Although species richness is the most commonly used measure to assess restoration success, it will neither reveal if the species are representative of the native community [27], nor distinguish between species with a robust population size and species on the edge of local extinction [77]. In contrast, using species composition as measurement of restoration outcome incorporates additional information, such as relative abundance [78]. Both the composition of all species and the grassland specialist species in our study changed significantly over time in the restored sites (from time T1 to T2). In contrast to plant richness and density, neither total species composition, nor grassland specialist composition in the restored sites were significantly different from the reference grasslands. Managed semi-natural grasslands typically harbour many species, but each species may have a low abundance, and therefore have little statistical impact in the species composition analyses. This could explain why species richness was higher in the reference sites than in the restored sites, whereas species composition did not differ. Since species richness includes species present as one single individual, and species composition considers frequency and relative abundance, analysing the latter can reveal species dominance shifts and may better indicate its resemblance with the community structure of reference sites [27]. The resemblance to reference habitats has been emphasized as a good measurement of restoration success (e.g. [26,27,39]), which is a goal partly accomplished by the similarities in species composition between these sites and reference grasslands.

A species composition similar to species-rich permanent semi-natural grasslands has only been reported by Schrautzer et al. [29], but several other studies have found promising effects in the right direction 5–15 years after restoration [28,30,40,41]. Helsen et al. [36] also found changes in species composition 15 years after restoration, where generalist species gradually were replaced by specialist species, indicating that similar results are achievable when given time.

### Local factors

Overall, the local factors had low, if any, effect on changes in species richness and composition. This could be due to the lack of precise data for some of our tested variables, but it could also be explained by the relatively small environmental differences between the sites. Nevertheless, the total species composition change was affected by abandonment time and fertilisation, while grassland area affected the specialist species composition, indicating that the local conditions could have an effect on some vegetation aspects following restoration.

Earlier studies show that a long time between abandonment and restoration, as well as high level of nutrients, have a negative effect on restoration potential in grasslands [37,79], resulting in few competitive species and loss of grassland specialists [80,81]. The abiotic and biotic conditions, such as species dominance, could have changed following abandonment [13,82] and thus affected the long-term development of species composition. The negative effects of fertilisers is well-studied in recreated grasslands on former arable fields (e.g. [70,83]), however restored degraded semi-natural grasslands are usually much lower in soil nutrients. Although, our study indicates that even very low amounts and small differences in degree of fertilisation could affect the species composition development following restoration (cf. [9]). However, fertilisation and the time between abandonment and restoration did not contribute to the significant change in grassland specialist composition over time in the restored sites, whereas the grassland area was significant. Long-lived grassland species have been found to have a positive dependency on habitat area [84], indicating that grassland area could be important to consider if restoration aims are related to grassland specialist species.

### What is considered restoration success?

Many management programmes today include some kind of ecological restoration, yet the definition of restoration success may not always be clear. Similarly, the reasons for restoration in our selected sites were in most cases not described. The goals were vague, relating mostly to the conservation and increase of biodiversity and cultural values, corresponding to the overarching goals for High Nature Value farmlands in EU. Having clear restoration goals is fundamental for the evaluation of restoration projects, irrespective of habitat type. The goals can be dynamic and have several possible end points [25], but they should be realistic [85]. Although this is often emphasized in restoration literature, setting up defined and quantitative goals in practice is still rather uncommon [20,85,86]. Regardless of restoration measure, it is important to evaluate restoration outcomes, especially in long-term follow-up studies. Short-term studies may reveal temporary trends, but the results might not be stable over time [18,87]. In this study, the species richness and density in the restored grasslands did not resemble reference conditions even after 12–20 years, while composition including all species as well as grassland specialists was similar. This indicates that different diversity measurements may lead to different outcomes, and thus different conclusions, which ultimately stress the importance of well-defined initial goals.

While broad overarching goals are important for habitat restoration in general there is still a need for measurable goals that can be evaluated through follow-up studies. These goals may specifically target different aspects of biodiversity conservation or be of interest for recreational or historical reasons. If the former is the main goal, we suggest that data of species frequency should be collected to follow the development of species composition over time, since the actual diversity could be missed when using only species richness or red-listed species [36,88]. Although species richness may increase, it is unclear if the additional species are habitat specialists or just a result of heterogeneity following restoration. Congruent with studies on grassland creation (e.g. [10,89]), our results suggest that restoration of a specific habitat type should include goals where species composition is analysed and compared to references of the desired state.

Even if the restoration of semi-natural grasslands is seen as successful, it still depends on continued management to maintain high biodiversity [35,90]. Since grazing on temporary grasslands (e.g. former arable fields) is more economical beneficial than grazing on species-rich pastures, these habitats are vulnerable to socio-economic changes [91]. The contract period for grassland restoration in European agri-environmental schemes is only 5–6 years [92], which could be a problem for the long-term management needed in these types of habitats [93].

## Conclusions

We have shown that the biodiversity measurements used to evaluate grassland restoration can have important implications for whether restoration might be considered a success. Although the over-all species richness increased, it was still higher in reference grasslands, even 12–20 years after restoration. Further, no significant changes were detected in number of grassland specialists or species density. The overall species composition and grassland specialist composition, on the other hand, had changed and now resembled reference grassland sites, indicating the importance of long-term follow-up studies. We suggest that resemblance to reference grasslands regarding species composition should be clearly included in the restoration aims for semi-natural grasslands, since it is a better indicator of restoration success. In that case these restorations could be considered successful, even though species richness did not resemble reference conditions. Having well defined restoration goals related to clear biological measures is hence the key to reach and evaluate restoration success.

## Supporting Information

S1 Appendix Plant species found in the restored and reference semi-natural grasslands. Classification of species into ‘Semi-natural grassland specialist species’ (S) or ‘Non-specialist’ (N). Mean abundance in reference sites at T2 (Time 2, i.e. year 2012) and restored sites at T1 (Time 1, i.e. year 2001) and T2. Differences in species abundance in the restored sites between T1 and T2 indicated as increasing (+) or decreasing (-) abundance. Species contribution to the dissimilarity in over-all species composition (i.e. species that are primarily responsible for the observed differences) in the restored sites between T1 and T2 (in %). Nomenclature according to Krok and Almquist.
(DOCX)
