# Supplementary material for: Long Term Positive Effect of Grassland Restoration on Plant Diversity - Success or Not?
Source: PLoS One. 2016 May 19;11(5):e0155836. doi: 10.1371/journal.pone.0155836 (PMC4873139; doi:10.1371/journal.pone.0155836)
Supplement: S1 Appendix — Classification of species into ‘Semi-natural grassland specialist species’ (S) or ‘Non-specialist’ (N). Mean abundance in reference sites at T2 (Time 2, i.e. year 2012) and restored sites at T1 (Time 1, i.e. year 2001) and T2. Differences in species abundance in the restored sites between T1 and T2 indicated as increasing (+) or decreasing (-) abundance. Species contribution to the dissimilarity in over-all species composition (i.e. species that are primarily responsible for the observed differences) in the restored sites between T1 and T2 (in %). Nomenclature according to Krok and Almquist. (DOCX) [file pone.0155836.s001.docx]

**S1 Appendix. Plant species found in the restored and reference semi-natural grasslands.** Classification of species into ‘Semi-natural grassland specialist species’ (S) or ‘Non-specialist’ (N). Mean abundance in reference sites at T2 (Time 2, i.e. year 2012) and restored sites at T1 (Time 1, i.e. year 2001) and T2. Differences in species abundance in the restored sites between T1 and T2 indicated as increasing (+) or decreasing (-) abundance. Species contribution to the dissimilarity in over-all species composition (i.e. species that are primarily responsible for the observed differences) in the restored sites between T1 and T2 (in %). Nomenclature according to Krok and Almquist[94].

| Species | Specialist (S) /Non-specialist (N) | Mean abund. Reference T2 | Mean abund. Restored T1 | Mean abund. Restored T2 | Diff. Restored T1 –T2 | Contrib. to dissim. (%) |
| --- | --- | --- | --- | --- | --- | --- |
| *Acer platanoides* | N | 0 | 0,022801 | 0,015625 | - | 0,2408 |
| *Achillea millefolium* | N | 5,9397792 | 6,3504 | 5,1984 | - | 0,8607 |
| *Achillea ptarmica* | N | 0 | 0,003906 | 0 | - | 0,05639 |
| *Aegopodium podagraria* | N | 0 | 0,0196 | 0,022801 | + | 0,2677 |
| *Agrimonia eupatoria* | S | 0 | 0,007815 | 0,015625 | + | 0,1726 |
| *Agrostis capillaris* | N | 3,5827688 | 9,2416 | 2,4336 | - | 1,443 |
| *Agrostis gigantea* | N | 0 | 0,003906 | 0 | - | 0,0634 |
| *Agrostis stolinifera* | N | 0 | 0,023409 | 0 | - | 0,1553 |
| *Agrostis vinealis* | S | 0 | 0 | 0,022801 | + | 0,1388 |
| *Ajuga pyramidalis* | S | 0,72 | 0,015625 | 0,314721 | + | 0,5711 |
| *Alchemilla glabra* | N | 0 | 0,207936 | 0,045369 | - | 0,5161 |
| *Alchemilla glaucescens* | S | 2,4286203 | 1,9044 | 0 | - | 1,283 |
| *Alchemilla monticola* | S | 2,5890379 | 0,3025 | 1,1025 | + | 0,9246 |
| *Alchemilla subcrenata* | N | 0,5329822 | 0 | 0,657721 | + | 0,7848 |
| *Allium oleraceum* | S | 0 | 0,015625 | 0,015625 | 0 | 0,195 |
| *Alopecurus pratensis* | N | 0 | 0,045369 | 0,214369 | + | 0,4925 |
| *Anemone nemorosa* | N | 2,0771896 | 0,023409 | 1,21 | + | 1,064 |
| *Antennaria dioica* | S | 0,36 | 0,007815 | 0,003906 | - | 0,1241 |
| *Anthoxanthum odoratum* | S | 7,8790264 | 1,3689 | 2,2801 | + | 1,044 |
| *Anthriscus sylvestris* | N | 0,36 | 3,4596 | 2,4336 | - | 0,7976 |
| *Anthyllis vulneraria* | S | 0,04 | 0 | 0 | NA | NA |
| *Arabidopsis thaliana* | N | 0 | 0,011664 | 0 | - | 0,1099 |
| *Arabis hirsuta* | S | 0 | 0 | 0,003906 | + | 0,06273 |
| *Arctium tomentosum* | N | 0 | 0,007815 | 0 | - | 0,094 |
| *Arrhenatherum elatius* | N | 0,12 | 0 | 0,035344 | + | 0,188 |
| *Asperula tingtoria* | N | 0,12 | 0 | 0 | NA | NA |
| *Astragalus glycyphyllos* | N | 0,08 | 0 | 0 | NA | NA |
| *Bellis perennis* | S | 0 | 0 | 0,003906 | + | 0,05586 |
| *Betula pendula* | N | 1,1725483 | 0,091204 | 0,239121 | + | 0,5459 |
| *Bistorta vivipara* | S | 1,1725483 | 0,003906 | 0 | - | 0,05955 |
| *Botrychium lunaria* | S | 0 | 0 | 0,003906 | + | 0,05108 |
| *Briza media* | S | 5,9143673 | 0,295936 | 0,190096 | - | 0,645 |
| *Bromus hordeaceus* | N | 0,04 | 0 | 0,003906 | + | 0,05027 |
| *Bromus inermis* | N | 0,48 | 0 | 0,022801 | + | 0,1388 |
| *Calamagrostis arundinacea* | N | 0 | 0,003906 | 0,143641 | + | 0,3589 |
| *Calluna vulgaris* | N | 0,64 | 0,003906 | 0,045369 | + | 0,2278 |
| *Campanula persicifolia* | N | 1,5555418 | 0,007815 | 0,835396 | + | 0,8312 |
| *Campanula rotundifolia* | S | 3,866589 | 1,7956 | 1,5876 | - | 0,9638 |
| *Capsella bursa-pastoris* | N | 0 | 0,003906 | 0,003906 | 0 | 0,1244 |
| *Cardamine pratensis* | N | 0 | 0,003906 | 0 | - | 0,05216 |
| *Carex diandra* | N | 0,04 | 0 | 0 | NA | NA |
| *Carex disticha* | N | 0,04 | 0 | 0 | NA | NA |
| *Carex echinata* | N | 0,04 | 0 | 0,003906 | + | 0,06273 |
| *Carex ericetorum* | S | 0,04 | 0 | 0 | NA | NA |
| *Carex hirta* | N | 0,04 | 0,112896 | 0,159201 | + | 0,5879 |
| *Carex hostiana* | S | 1,7215948 | 0 | 0,022801 | + | 0,1651 |
| *Carex leporina* | S | 0,5571281 | 0,035344 | 0,4096 | + | 0,5774 |
| *Carex montana* | S | 0 | 0,011664 | 0 | - | 0,1034 |
| *Carex nigra* | N | 0,8319184 | 0 | 0 | NA | NA |
| *Carex pallescens* | S | 2,3913844 | 0,595984 | 0,470596 | - | 0,905 |
| *Carex panicea* | S | 1,0259644 | 0 | 0,007815 | + | 0,0788 |
| *Carex pilulifera* | S | 0,08 | 0,132496 | 0,003906 | - | 0,362 |
| *Carex spicata* | S | 0,2331371 | 0,143641 | 0,815409 | + | 0,8636 |
| *Carex vesicaria* | N | 0,04 | 0 | 0 | NA | NA |
| *Carlina vulgaris* | S | 0,08 | 0 | 0 | NA | NA |
| *Carum carvi* | S | 0,6298387 | 0,199809 | 0,046656 | - | 0,5541 |
| *Centaurea jacea* | N | 2,062981 | 0,7396 | 0,772641 | + | 0,9375 |
| *Centaurea scabiosa* | N | 0,2985641 | 0 | 0 | NA | NA |
| *Cerastium fontanum* | N | 2,4314451 | 1,7161 | 1,5625 | - | 1,013 |
| *Chenopodium album* | N | 0 | 0 | 0,015625 | + | 0,1345 |
| *Chrysosplenium alternifolium* | N | 0 | 0,011664 | 0 | - | 0,1099 |
| *Cirsium acaule* | S | 0,2 | 0 | 0 | NA | NA |
| *Cirsium arvense* | N | 0,04 | 0,177241 | 0,054289 | - | 0,4873 |
| *Cirsium palustre* | N | 0,16 | 0 | 0 | NA | NA |
| *Cirsium vulgare* | N | 0 | 0,015625 | 0,015625 | 0 | 0,2047 |
| *Convallaria majalis* | N | 0,7919184 | 0,022801 | 0,070225 | + | 0,3048 |
| *Convolvulus arvensis* | N | 0 | 0 | 0,003906 | + | 0,06723 |
| *Corylus avellana* | N | 0 | 0 | 0,087616 | + | 0,287 |
| *Crataegus monogyna* | N | 0 | 0 | 0,003906 | + | 0,05572 |
| *Cynosorus cristatus* | S | 1,1986652 | 0 | 0 | NA | NA |
| *Dactylis glomerata* | N | 1,3838319 | 2,25 | 2,7556 | + | 1,252 |
| *Dactylorhiza latifolia* | N | 0 | 0,003906 | 0,022801 | + | 0,1662 |
| *Dactylorhiza maculata* | S | 0,12 | 0,229441 | 0,022801 | - | 0,4898 |
| *Danthonia decumbens* | S | 0,7919184 | 0 | 0 | NA | NA |
| *Deschampsia cespitosa* | N | 3,3461661 | 1,3689 | 3,9601 | + | 1,189 |
| *Deschampsia flexuosa* | N | 4,723753 | 0,321489 | 3,1684 | + | 1,309 |
| *Dianthus deltoides* | S | 0 | 0,022801 | 0,022801 | 0 | 0,2601 |
| *Dryopteris filix-mas* | N | 0 | 0 | 0,007815 | + | 0,09517 |
| *Eleocharis uniglumis* | N | 0,04 | 0 | 0 | NA | NA |
| *Elytrigia repens* | N | 0,04 | 0,564001 | 0,380689 | - | 0,9864 |
| *Equisetum arvense* | N | 0,16 | 0 | 0,003906 | + | 0,06949 |
| *Equisetum pratense* | N | 0 | 0,029241 | 0 | - | 0,1599 |
| *Equisetum sylvaticum* | N | 0,04 | 0 | 0 | NA | NA |
| *Euphrasia nemorosa* | S | 0 | 0,003906 | 0 | - | 0,05391 |
| *Festuca brevipila* | N | 0 | 0,093636 | 0 | - | 0,3104 |
| *Festuca ovina* | S | 6,9403219 | 7,29 | 2,9241 | - | 1,183 |
| *Festuca pratensis* | N | 1,1587801 | 1,2996 | 1,6384 | + | 1,054 |
| *Festuca rubra* | S | 0 | 0,035344 | 0,003906 | - | 0,2078 |
| *Filipendula ulmaria* | N | 0,16 | 0,045369 | 0,093025 | + | 0,4295 |
| *Filipendula vulgaris* | N | 2,8075027 | 1,4884 | 1,8225 | + | 1,122 |
| *Fragaria vesca* | N | 1,2367566 | 0,693889 | 1,96 | + | 1,028 |
| *Fragaria viridis* | S | 0,28 | 0,007815 | 0 | - | 0,07625 |
| *Fraxinus excelsior* | N | 0,04 | 0,126736 | 0,143641 | + | 0,5886 |
| *Galium album* | N | 0 | 0,1521 | 0,0625 | - | 0,5201 |
| *Galium aparine* | N | 0,04 | 0 | 0,067081 | + | 0,2731 |
| *Galium boreale* | N | 1,7725483 | 1,3456 | 0,970225 | - | 1,015 |
| *Galium saxatile* | S | 0 | 0,015625 | 0 | - | 0,1101 |
| *Galium spurium* | N | 0,04 | 0 | 0,003906 | + | 0,06729 |
| *Galium uliginosum* | S | 0,2985641 | 0,054289 | 0 | - | 0,2283 |
| *Galium verum* | N | 1,6638367 | 3,6481 | 2,4964 | - | 0,8863 |
| *Geranium pusillum* | N | 0 | 0 | 0,015625 | + | 0,1186 |
| *Geranium pyrenaicum* | N | 0 | 0 | 0,003906 | + | 0,06273 |
| *Geranium robertianum* | N | 0 | 0 | 0,003906 | + | 0,06273 |
| *Geranium sanguineum* | N | 0 | 0 | 0,058081 | + | 0,2076 |
| *Geranium sylvaticum* | N | 0,7919184 | 0,1225 | 0,992016 | + | 0,9711 |
| *Geum rivale* | N | 1,9655859 | 0,8281 | 0,850084 | + | 0,9496 |
| *Geum urbanum* | N | 0,04 | 0,007815 | 0,076176 | + | 0,3187 |
| *Glechoma hederacea* | N | 0 | 0 | 0,029241 | + | 0,1498 |
| *Glyceria fluitans* | N | 0 | 0 | 0,003906 | + | 0,06723 |
| *Gnaphalium sylvaticum* | N | 0 | 0 | 0,003906 | + | 0,05623 |
| *Helianthemum nummularium* | S | 0,8956922 | 0,057121 | 0,140625 | + | 0,4487 |
| *Helictotrichon pratense* | N | 2,4534975 | 0,0625 | 0,872356 | + | 0,8708 |
| *Helictotrichon pubescens* | N | 0 | 0,432964 | 0,223729 | - | 0,7254 |
| *Hepatica nobilis* | N | 0,08 | 0,1225 | 0,298116 | + | 0,6559 |
| *Hieracium L. sect. Hieracium* | N | 0 | 0,015625 | 0,003906 | - | 0,1559 |
| *Hieracium L. sect. Oreada* | N | 0,08 | 0 | 0 | NA | NA |
| *Hieracium L. sect. Vulgata* | N | 0,6876603 | 0,015625 | 0,0625 | + | 0,2993 |
| *Hieracium lactocella* | S | 0 | 0,015625 | 0 | - | 0,1133 |
| *Hieracium pilosella* | S | 2,4919289 | 0,64 | 1,4884 | + | 0,9273 |
| *Hieracium umbellatum* | N | 0 | 0,011664 | 0,003906 | - | 0,1427 |
| *Hypericum maculatum* | N | 0,12 | 0,4761 | 0,363609 | - | 0,8616 |
| *Hypericum perforatum* | S | 0,8633202 | 0,698896 | 0,772641 | + | 0,9394 |
| *Hypochaeris macalata* | S | 0,08 | 0 | 0 | NA | NA |
| *Juncus compressus* | S | 0,04 | 0 | 0 | NA | NA |
| *Juncus conglomeratus* | N | 0,08 | 0,003906 | 0,007815 | + | 0,1272 |
| *Juncus effusus* | N | 0,04 | 0,003906 | 0,015625 | + | 0,1621 |
| *Juncus filiformis* | S | 0,04 | 0 | 0 | NA | NA |
| *Juniperus communis* | N | 0,04 | 0,003906 | 0,007815 | + | 0,1301 |
| *Knautia arvensis* | N | 0,5571281 | 0,107584 | 0,226576 | + | 0,5639 |
| *Laserpitium latifolium* | N | 0,04 | 0 | 0 | NA | NA |
| *Lathyrus linifolius* | S | 2,1988225 | 2,4025 | 1,7956 | - | 1,024 |
| *Lathyrus pratensis* | N | 2,062981 | 1,5129 | 2,2201 | + | 1,065 |
| *Lathyrus vernus* | N | 0,04 | 0 | 0,007815 | + | 0,079 |
| *Leontodon autumnalis* | S | 0,6876603 | 0,434281 | 0,308025 | - | 0,8174 |
| *Leontodon hispidus* | S | 0 | 0,003906 | 0 | - | 0,0634 |
| *Leucanthemum vulgare* | S | 4,4599887 | 0,140625 | 0,565504 | + | 0,7854 |
| *Linum catharticum* | S | 1,7401589 | 0,007815 | 0,022801 | + | 0,1862 |
| *Lolium perenne* | N | 0 | 0 | 0,003906 | + | 0,05623 |
| *Lotus corniculatus* | S | 4,8959172 | 0,855625 | 0,879844 | + | 1,02 |
| *Luzula campestris* | S | 4,9442967 | 0,029241 | 2,1904 | + | 1,327 |
| *Luzula multiflora* | N | 1,5588225 | 0,931225 | 0,184041 | - | 0,913 |
| *Luzula pallescens* | S | 0 | 0 | 0,003906 | + | 0,06729 |
| *Luzula pilosa* | N | 0,6876603 | 0,087616 | 0,205209 | + | 0,5662 |
| *Lychnis flos-cuculi* | S | 0 | 0 | 0,003906 | + | 0,05572 |
| *Lychnis viscaria* | S | 0 | 0,003906 | 0,058081 | + | 0,2519 |
| *Lysimachia vulgaris* | N | 0,04 | 0 | 0 | NA | NA |
| *Maianthemum bifolium* | N | 0 | 0,003906 | 0 | - | 0,05791 |
| *Malus sylvestris* | N | 0 | 0,003906 | 0 | - | 0,05791 |
| *Matricaria recutita* | N | 0 | 0,003906 | 0 | - | 0,07476 |
| *Medicago lupulina* | S | 0,28 | 0 | 0 | NA | NA |
| *Melampyrum cristatum* | S | 0 | 0 | 0,011664 | + | 0,09676 |
| *Melampyrum pratense* | N | 0,08 | 0 | 0,003906 | + | 0,05572 |
| *Melampyrum sylvaticum* | N | 0,32 | 0,003906 | 0,015625 | + | 0,1765 |
| *Melica nutans* | N | 0 | 0,022801 | 0,003906 | - | 0,1736 |
| *Mentha arvensis* | N | 0,04 | 0 | 0 | NA | NA |
| *Milium effusum* | N | 0 | 0 | 0,003906 | + | 0,06729 |
| *Moehringia trinervia* | N | 0 | 0,015625 | 0 | - | 0,1269 |
| *Mycelis muralis* | N | 0 | 0 | 0,007815 | + | 0,09517 |
| *Myosotis arvensis* | N | 0 | 0,003906 | 0 | - | 0,06229 |
| *Myosotis scorpioides* | S | 0,04 | 0 | 0 | NA | NA |
| *Myosotis sylvatica* | N | 0 | 0 | 0,007815 | + | 0,0711 |
| *Nardus stricta* | S | 0 | 0 | 0,029241 | + | 0,1573 |
| *Origanum vulgare* | N | 0 | 0,003906 | 0,023409 | + | 0,1683 |
| *Orthilia secunda* | N | 0 | 0 | 0,003906 | + | 0,06729 |
| *Oxalis acetosella* | N | 0 | 0,035344 | 0,051984 | + | 0,3686 |
| *Paris quadrifolia* | N | 0 | 0 | 0,007815 | + | 0,09517 |
| *Phleum phleoides* | N | 0,12 | 0 | 0 | NA | NA |
| *Phleum pratense* | N | 0,3959592 | 1,9044 | 1,6641 | - | 1,142 |
| *Picea abies* | N | 0 | 0 | 0,003906 | + | 0,06729 |
| *Pilosella dubia* | N | 0 | 0 | 0,003906 | + | 0,05586 |
| *Pimpinella saxifraga* | S | 2,7861758 | 1,1449 | 1,1236 | - | 1,088 |
| *Pinus sylvestris* | N | 0,04 | 0,003906 | 0,045369 | + | 0,2424 |
| *Plantago lanceolata* | S | 6,5664669 | 2,0164 | 2,0449 | + | 1,23 |
| *Plantago major* | N | 0,04 | 0,335241 | 0,619369 | + | 0,9604 |
| *Plantago media* | S | 0,32 | 0,003906 | 0,003906 | 0 | 0,1225 |
| *Platanthera bifolia* | S | 0,04 | 0 | 0,003906 | + | 0,05586 |
| *Poa annua* | N | 0,2331371 | 0,076176 | 0,514089 | + | 0,7169 |
| *Poa compressa* | N | 0 | 0,054289 | 0 | - | 0,2444 |
| *Poa nemoralis* | N | 0,04 | 0 | 0,114244 | + | 0,3485 |
| *Poa palustris* | N | 0,12 | 0,003906 | 0,051984 | + | 0,2668 |
| *Poa pratensis* | N | 3,2857797 | 5,2441 | 5,9536 | + | 0,7286 |
| *Poa remota* | N | 0,04 | 0 | 0 | NA | NA |
| *Poa supina* | S | 0 | 0,003906 | 0 | - | 0,06344 |
| *Polygala vulgaris* | S | 2,717645 | 0,097969 | 0,813604 | + | 0,8056 |
| *Polygonatum odoratum* | N | 0 | 0,003906 | 0,035344 | + | 0,1956 |
| *Polygonum aviculare* | S | 0 | 0,007815 | 0,003906 | - | 0,1624 |
| *Polypodium vulgare* | N | 0 | 0 | 0,003906 | + | 0,05027 |
| *Populus tremula* | N | 0 | 0,003906 | 0,003906 | 0 | 0,111 |
| *Potentilla anserina* | S | 0,04 | 0,003906 | 0 | - | 0,06647 |
| *Potentilla argentea* | S | 0 | 0,003906 | 0,045369 | + | 0,2427 |
| *Potentilla erecta* | N | 5,0999213 | 0,777924 | 1,0201 | + | 0,9287 |
| *Potentilla reptans* | N | 0 | 0,162409 | 0,022801 | - | 0,4407 |
| *Potentilla tabernaemontani* | S | 0,04 | 0,007815 | 0 | - | 0,08972 |
| *Primula veris* | S | 1,0593615 | 0,407044 | 1,5625 | + | 1,02 |
| *Prunella vulgaris* | N | 4,807911 | 0,425104 | 0,494209 | + | 0,8364 |
| *Prunus spinosa* | N | 0,04 | 0,076176 | 0,045369 | - | 0,3625 |
| *Pteridium aquilinum* | N | 0,2331371 | 0,067081 | 0,205209 | + | 0,5478 |
| *Pulmonaria obscura* | N | 0 | 0 | 0,003906 | + | 0,06729 |
| *Pulsatilla vulgaris* | S | 0,24 | 0 | 0,003906 | + | 0,0653 |
| *Pyrola rotundifolia* | N | 0 | 0,003906 | 0 | - | 0,05216 |
| *Quercus robur* | N | 0,04 | 0,0841 | 0,199809 | + | 0,5721 |
| *Ranunculus acris* | S | 4,6342366 | 6,1009 | 4,4944 | - | 0,9908 |
| *Ranunculus auricomus* | N | 1,28 | 0,147456 | 0,824464 | + | 0,7781 |
| *Ranunculus bulbosus* | S | 0,5571281 | 0,035344 | 0,1681 | + | 0,4448 |
| *Ranunculus cassubicus* | N | 0 | 0 | 0,045369 | + | 0,1961 |
| *Ranunculus polyanthemos* | S | 0 | 0 | 0,054289 | + | 0,2174 |
| *Ranunculus repens* | N | 0,04 | 0,589824 | 0,279841 | - | 0,6988 |
| *Rhinanthus minor* | S | 0,36 | 0 | 0,124609 | + | 0,2982 |
| *Rhinanthus serotinus* | S | 2,2556763 | 0 | 0,022801 | + | 0,1486 |
| *Rosa canina* | N | 0,04 | 0,003906 | 0,022801 | + | 0,1798 |
| *Rosa dumalis* | N | 0,16 | 0,015625 | 0,147456 | + | 0,3894 |
| *Rosa majalis* | N | 0 | 0,003906 | 0 | - | 0,05971 |
| *Rosa villosa* | N | 0,04 | 0,035344 | 0 | - | 0,1793 |
| *Rubus idaeus* | N | 0 | 0,251001 | 0,265225 | + | 0,6867 |
| *Rubus saxatilis* | N | 0,04 | 0,003906 | 0,045369 | + | 0,2374 |
| *Rubus sect. Corylifolii* | N | 0 | 0,003906 | 0 | - | 0,05051 |
| *Rumex acetosa* | N | 4,3798969 | 3,3489 | 3,3124 | - | 1,003 |
| *Rumex acetosella* | S | 0 | 0,097969 | 0,029241 | - | 0,4065 |
| *Rumex crispus* | N | 0 | 0,003906 | 0,003906 | 0 | 0,1244 |
| *Sagina procumbens* | S | 0 | 0,035344 | 0 | - | 0,1848 |
| *Saxifraga granulata* | S | 0 | 0,007815 | 0 | - | 0,07143 |
| *Scleranthus annus* | N | 0 | 0,003906 | 0 | - | 0,07476 |
| *Scorzonera humilis* | S | 0,6929822 | 0 | 0,003906 | + | 0,05027 |
| *Scutellaria galericulata* | N | 0,04 | 0 | 0 | NA | NA |
| *Sedum acre* | N | 0 | 0,003906 | 0,003906 | 0 | 0,118 |
| *Sedum album* | N | 0 | 0,003906 | 0 | - | 0,06344 |
| *Sedum annum* | N | 0 | 0,003906 | 0 | - | 0,07476 |
| *Sedum rupestre* | N | 0 | 0,003906 | 0,003906 | 0 | 0,09538 |
| *Sedum sexangulare* | N | 0 | 0,003906 | 0 | - | 0,06344 |
| *Sedum telephium* | N | 0 | 0,015625 | 0,029241 | + | 0,2361 |
| *Senecio sylvaticus* | N | 0 | 0 | 0,003906 | + | 0,0653 |
| *Sesleria uliginosa* | N | 0 | 0,003906 | 0 | - | 0,05639 |
| *Solidago virgaurea* | N | 0 | 0 | 0,003906 | + | 0,06723 |
| *Sorbus aucuparia* | N | 0 | 0,087616 | 0,25 | + | 0,6163 |
| *Spergularia rubra* | N | 0 | 0 | 0,003906 | + | 0,05027 |
| *Stachys palustris* | N | 0 | 0 | 0,003906 | + | 0,06304 |
| *Stellaria graminea* | N | 3,3228792 | 4,8841 | 3,7636 | - | 0,5951 |
| *Stellaria holostea* | N | 0 | 0 | 0,003906 | + | 0,06729 |
| *Stellaria longifolia* | N | 0 | 0 | 0,003906 | + | 0,06729 |
| *Stellaria media* | N | 0 | 0,035344 | 0,091204 | + | 0,4204 |
| *Succisa pratensis* | S | 0,6929822 | 0,529984 | 0,199809 | - | 0,7454 |
| *Tanacetum vulgare* | N | 0 | 0,265225 | 0 | - | 0,5243 |
| *Taraxacum sect. Vulgaria* | N | 2,5985214 | 3,8025 | 3,3124 | - | 0,9488 |
| *Thalictrum flavum* | N | 0,08 | 0 | 0,007815 | + | 0,0831 |
| *Thlapsi caernlescens* | N | 0 | 0,015625 | 0,132496 | + | 0,422 |
| *Thymus serpyllum* | S | 0 | 0,003906 | 0 | - | 0,05391 |
| *Tragopogon pratensis* | N | 0 | 0,015625 | 0,003906 | - | 0,1599 |
| *Trientalis europaea* | N | 0 | 0 | 0,003906 | + | 0,06723 |
| *Trifolium arvense* | N | 0 | 0 | 0,003906 | + | 0,05027 |
| *Trifolium aureum* | N | 0 | 0,003906 | 0,007815 | + | 0,1245 |
| *Trifolium medium* | N | 3,8025412 | 0 | 2,7889 | + | 1,572 |
| *Trifolium montanum* | S | 0,04 | 0 | 0 | NA | NA |
| *Trifolium pratense* | N | 3,0919816 | 4,9284 | 2,1609 | - | 1,15 |
| *Trifolium repens* | S | 8,1310736 | 7,0225 | 5,3361 | - | 0,8919 |
| *Urtica dioica* | N | 0 | 0,022801 | 0,114244 | + | 0,4212 |
| *Vaccinium myrtillus* | N | 0,36 | 0,182329 | 0,248004 | + | 0,61 |
| *Vaccinium vitis-idaea* | N | 0,2985641 | 0,076176 | 0,199809 | + | 0,5305 |
| *Veronica arvensis* | N | 0 | 0 | 0,022801 | + | 0,1449 |
| *Veronica chamaedrys* | S | 4,1650171 | 6,25 | 4,0804 | - | 0,7161 |
| *Veronica officinalis* | S | 2,2285125 | 1,1025 | 3,2761 | + | 0,9576 |
| *Veronica serpyllifolia* | N | 0 | 1,0609 | 0,107584 | - | 0,949 |
| *Veronica spicata* | S | 0 | 0,007815 | 0,003906 | - | 0,1163 |
| *Veronica verna* | S | 0 | 0,003906 | 0 | - | 0,07476 |
| *Vicia cassubica* | N | 0 | 0,003906 | 0 | - | 0,05391 |
| *Vicia cracca* | N | 2,062981 | 2,25 | 0,793881 | - | 1,019 |
| *Vicia lathyroides* | S | 0 | 0,003906 | 0 | - | 0,05051 |
| *Vicia sepium* | N | 0,04 | 0,164025 | 0,732736 | + | 0,92 |
| *Vicia sylvatica* | N | 0 | 0 | 0,007815 | + | 0,0788 |
| *Vicia tetrasperma* | N | 0 | 0 | 0,248004 | + | 0,4812 |
| *Vincetoxicum hirundinaria* | N | 0 | 0,003906 | 0,007815 | + | 0,1162 |
| *Viola canina* | S | 2,3410723 | 1,96 | 2,1025 | + | 0,8707 |
| *Viola hirta* | N | 0,2331371 | 0,067081 | 0,067081 | 0 | 0,3988 |
| *Viola reichenbachiana* | N | 0 | 0 | 0,035344 | + | 0,1806 |
| *Viola riviniana* | N | 0,5571281 | 0,015625 | 0,498436 | + | 0,6795 |
| *Viola tricolor* | S | 0,08 | 0 | 0,035344 | + | 0,1641 |
| (Unknown species) | (NA) | 0 | 0,218089 | 0 | - |  |
|  |  |  |  |  |  |  |

**References**

94. Krok T, Almquist S. Svensk flora: Fanerogamer och kärlkryptogamer. Sweden [in Swedish]; 2012.
